# Response to ‘Comment on ‘Domestic light at night and breast cancer risk: a prospective analysis of 105 000 UK women in the Generations Study”

**DOI:** 10.1038/s41416-018-0344-y

**Published:** 2018-12-25

**Authors:** Louise E. Johns, Michael E. Jones, Minouk J. Schoemaker, Emily McFadden, Alan Ashworth, Anthony J Swerdlow

**Affiliations:** 1 Division of Genetics and Epidemiology, The Institute of Cancer Research, London, SM2 5NG UK; 2 Division of Breast Cancer Research, The Institute of Cancer Research, London, SW3 6JB UK; 3 Breast Cancer Now Research Centre, The Institute of Cancer Research, London, SW3 6JB UK; 4 Division of Molecular Pathology, The Institute of Cancer Research, London, SW7 3RP UK; 5 Present Address: Nuffield Department of Primary Care Health Sciences, University of Oxford, Oxford, OX2 6GG UK; 6 Present Address: UCSF Helen Diller Family Comprehensive Cancer Center, San Francisco, CA 94158 USA

Dear Editor,

We thank Drs Kyba and Spitschan for their thoughtful comments.^1^ We agree that the responses we received^2^ from our study participants on extent of light at night in bedrooms are a complex reflection of actual visual ability, subjective judgement of visual ability, and recall and generalisation of this. It may indeed be possible to develop more-valid questions, especially, as Drs Kyba and Spitschan say, if these can be evaluated against measured light in the bedroom. However, it will not be simple since, for instance, actual light at night will vary by season and according to the weather.

## References

[1] Kyba CCM, Spitschan M (2018). Comment on ‘Domestic light at night and breast cancer risk: a prospective analysis of 105 000 UK women in the Generations Study. Br. J. Cancer.

[2] Johns LE (2018). Domestic light at night and breast cancer risk: a prospective analysis of 105 000 UK women in the Generations Study. Br. J. Cancer.

